# Communicative performance and vocabulary domain in preschool preterm infants

**DOI:** 10.1590/1678-7757-2017-0186

**Published:** 2018-07-06

**Authors:** Dionísia Aparecida Cusin LAMÔNICA, Caroline Kauffman BECARO, Aline Cabral BORBA, Luciana de Paula MAXIMINO, Aline Roberta Aceituno da COSTA, Camila da Costa RIBEIRO

**Affiliations:** 1 Universidade de São Paulo, Faculdade de Odontologia de Bauru, Departamento de Fonoaudiologia, Bauru, São Paulo, Brasil.

**Keywords:** Preterm infant, Child development, Vocabulary, Preschool child

## Abstract

**Objective:**

The objective of this study was to evaluate and compare the performance of children in preschool age who were born premature and term, without neurological injury, regarding receptive and expressive language skills, and to reflect on the importance of these skills for performance in preschool.

**Materials and Methods:**

Two groups named Preterm Group and Comparison Group, each composed by 40 children, as well as 80 legal representatives (mothers) and 80 teachers of the participants. To pair the groups, we considered chronological age (months), sex, educational level, type of school (public or private) and socioeconomic status. To assess the groups we used structured and semi-structured Observation of Communicative Behavior and applied the ABFW Child Language Test - Part B-Vocabulary and the Peabody Picture Vocabulary Test. To assess the legal representatives we applied an anamnesis questionnaire and the MacArthur Communicative Development Inventory. The assessment of the teachers consisted of the MacArthur Communicative Development Inventory and a Student Assessment Protocol developed by the authors.

**Results:**

For the observation of communicative behavior, the categories with the highest losses were: narrative, maintaining dialogic activities and attention difficulties. In the ABFW Child Language Test and Peabody Picture Vocabulary Test there were statistically significant differences. In the MacArthur Communicative Development Inventory there were statistically significant differences in expressive vocabulary, but no differences in receptive vocabulary, for both the mothers and the teachers.

**Conclusion:**

Children born prematurely with low risk of neurological sequelae in preschool age may have greater difficulties in linguistic performance than their peers born to term.

## Introduction

Prematurity is a relevant condition in public health, due to the variability in developmental trajectories of children, it also may cause negative academic and social-emotional repercussions^22^.

The World Health Organization (WHO) defines preterm birth as any birth before 37 completed weeks of gestation, this can be further subdivided based on gestational age: extremely preterm (<28 weeks), very preterm (28 – <32 weeks) and moderate to late preterm (32 – <37 weeks) completed weeks of gestation^19^.

Premature infants are born in a fragile condition, which often places them in a risk situation, not only regarding survival, but also possible deficits in the overall development, specifically, language and learning. This type of deficit can manifest later in life, such as in school age^13^ ^,^ ^14^ ^,^ ^17^ ^,^ ^18^ ^,^ ^21^ ^,^ ^25^.

Despite being widely recognized, the relation of this issue on school performance, as well as the nature of learning difficulties, are not yet fully understood^25^ ^,^ ^28^.

The development prognosis of preterm infants depends on the interaction of biological and environmental factors that affect the immature brain of the child. Several factors are reported as risk factors that increase the chances for changes in development, among them the presence of gestational and neonatal morbidities, gestational age, low and very low birth weight and neonatal and postnatal complications^6^ ^,^ ^21^. The greater the number of active risk factors, the greater the potential for developmental disorders.

Many researchers focus their studies on the developmental risks of extremely premature infants and individuals of very low birth weight; however, moderate and late preterm infants are also risk individuals for such changes.

Language is crucial for communication, academic performance and social function, therefore, it must be accompanied throughout child development^24^ ^,^ ^25^ ^,^ ^30^. Regarding the development of language, studies show that there is a high probability of developmental delay for both receptive and expressive processes for premature infants, or even that they punctuate relatively less in evaluations of these processes when compared to children born at term^3^ ^,^ ^5^ ^,^ ^11^ ^,^ ^12^ ^,^ ^16^ ^,^ ^20^ ^,^ ^22^ ^,^ ^28^ ^,^ ^29^. However, these aspects require further studies.

Difficulties in school learning can be related to language acquisition delays. These should be identified at an earlier age, even in the absence of evidence of brain injury^2^.

Studies on language development focus on the effects of prematurity on the performance of children in preschool and other school years, due to the possibility of difficulties in cognitive skills, particularly attention and executive functions, reduced performance in language on different linguistic levels, as well as behavioral problems^1^ ^,^ ^15^ ^,^ ^17^ ^,^ ^20^ ^,^ ^30^ ^,^ ^31^.

Given this context, the objective of this study was to evaluate and compare the performance of children in preschool age who were born premature and term, without neurological injury, regarding receptive and expressive language skills, and to reflect on the effects of these for performance in preschool.

## Materials and methods

This study was approved by the Research Ethics Committee (Protocol: 06902812.7.0000.5417). This is a cross-sectional analytical observational study. The study started after the legal representative of the child signed the Informed Consent Form.

The participants of the study were 40 premature children, constituting the Preterm Group (PG), and 40 children born at term constituting the Comparison Group (CG), as well as 80 legal representatives (mothers) and 80 teachers of the participating children.

### Inclusion criteria

Preterm group (PG): born with gestational age under 37 weeks; 5-minute Apgar score over 7; aged between four to five years and eleven months of chronological age; underwent hearing, visual and metabolism evaluations (congenital hypothyroidism, phenylketonuria, hemoglobinopathies) with normative results; no delay in neurodevelopment and no complaint of developmental changes from the interviewed mothers.

Comparison Group (CG): was born at term with a gestational age greater than 38 weeks, present typical development; be paired with the PG; underwent hearing, visual and metabolism evaluations with normative results.

We considered chronological age (in months), sex, educational level, type of school (public or private) and socioeconomic status to pair the groups. Each child in the CG was paired with a child from the PG, with the same characteristics, according to all variables described above.

The evaluation process of the parents consisted of the application of an anamnesis questionnaire and of the MacArthur Communicative Development Inventory (MCDI)^10^. The anamnesis questionnaire contained questions about the early life of the children, from pregnancy until the present age, regarding the complications, psychomotor development, language, self-care, family relationships and school records, and identification data consisting of maternal age, education level of the parents, among other information. Mothers also answered the Brazilian Economic Classification Criteria – Brazilian Criteria^2^, which consisted of information on the social class to later perform the pairing between the groups.

Teachers were contacted after the family accepted to participate in the study, they were informed about the objectives. After signing the informed consent form, we applied the MCDI^10^ and a Student Assessment Protocol. The protocol was developed by the authors to verify the perceptions of the teacher on the performance of their students, the protocol contains the following questions: Does the student regularly attend school? Does the child communicate well? Does he/she present learning problems? Does he/she have difficulty to maintain attention in academic activities? Does he/she present memory problems? Does he/she present behavior problems? Does he/she have good relations with other colleagues? Is he/she independent in class activities?

Children were excluded when their mothers and/or teachers did not want to participate.

The instrument used to assess the mothers and teachers was:

MacArthur Communicative Development Inventory (MCDI) - First words and gestures, Brazilian Portuguese adaptation^10^. We applied Section D, which consists of 421 items organized into 22 semantic categories. Ten of these semantic categories included nouns, verbs; qualities and attributes; pronouns; interrogative words; prepositions; locatives; quantifiers; words of time; states and articles. The participants (mothers and teachers) were asked which categories the children only understood (reception) and those that they understand and speak (reception and expression). This instrument is standardized for children aged from eight to sixteen months. However, in this study, it was used only as a check list of vocabulary.

The instruments used to evaluate the children were:

Observation of Communicative Behavior (OCB)^30^. The participants were placed in structured and semi-structured situations held during a session of about 50 minutes with playful and interactive activities. To verify their actions and interactions, they were offered concrete objects. These situations were filmed for analysis. We analyzed the communication skills of the participants, which include dialogic and conversational skills, communication functions, media, context of language, verbal comprehension, forms of manipulation and functional use of objects, symbolism, toy organization and imitation. Twenty three categories were analyzed: communicative intent; interaction with the evaluator; eye contact; vocalizations; production of words; production of sentences with more than 2 elements; respect for turn changes; starting dialogue; participation in dialogic activity; maintaining dialogue; narrative; understanding specific situations; understanding of abstract situations; performing simple orders; performing complex orders; symbolic play; attention span; interest in toys; function to inform; function to protest; function to request; function to offer; function to imitate. These communicative behavior analysis categories are calculated with the following criteria: 0 - not presented; 1 - presented in restricted situations of self-interest; 2 - presented in any situation. We used the sum of the categories after analyzing the footage for statistical analysis. Considering the total items and analysis criteria, the maximum sum is 46 points.

ABFW Child Language Test - Vocabulary Part B^4^. This instrument evaluates qualitatively and quantitatively the expressive vocabulary of children in nine conceptual fields: clothing, animals, food, transportation, furniture and utensils, professions, places, shapes and colors, toys and musical instruments. The responses were filmed and recorded in a specific protocol for later analysis. The participants are asked to designate each figure presented. We followed the rules proposed in the instruction manual for the analysis of the names of the usual words (UVD – correct appointment), non-designations (ND) and replacement processes (RP) – producing another word or functionality. These data were organized in a database for analysis. The initial analysis consisted of obtaining the mean of the usual vocabulary assignments, non-designations and replacement processes from each participant through the sum of the percentages obtained in each of these items and dividing by the total conceptual fields assessed.

Peabody Picture Vocabulary Test (PPVT)^9^. The objective of this test is to assess the lexical development in the receptive field, providing information on the receptive-hearing vocabulary. We followed the rules proposed in the instruction manual to establish the base and the ceiling of the responses.

### Characterization of casuistry

**Figure 1:**
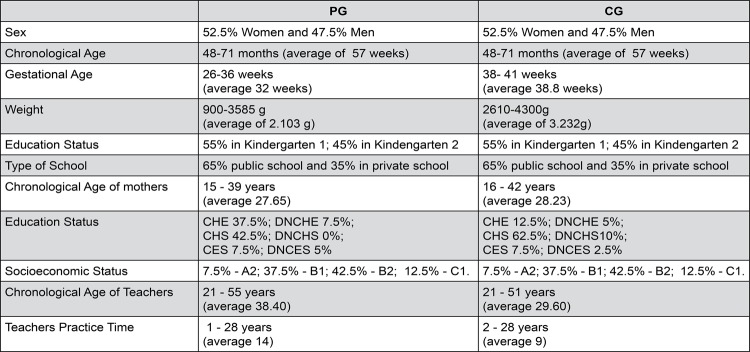
Characterization of casuistry

PG: Preterm Group; CG: Comparative Group; Kindergarten 1: children with age up to 5 years; Kindergarten 2: children with age above 5 years; CES: completed elementary school; DNCES: did not complete elementary school; CHS: completed high school; DNCHS: did not complete high school; CHE: completed higher education; DNCHE: did not complete higher education

Figure 1 shows the characterization of the casuistry.

Mothers reported that they received no medical diagnosis, and that the children did not attend appointments for any area of development and did not identify the development problems of their children.

For statistical analysis we used the mean, minimum and maximum values of descriptive analysis, we also used Student’s t-test when the groups had normal distribution and the Mann-Whitney test when at least one of the groups did not show normal distribution (p≤0.05). The Kolmogorov-Smirnov test was used to test normality. The analysis was performed using the Statistical Package for Social Science software (SPSS), version 17.0.

## Results

Tables 1, 2, 3 and 4 present the mean, minimum and maximum values, standard deviation and *p* value on the comparison between the PG and CG.

**Table 1:** Results of the OCB instrument of the PG compared to the CG

Group	Mean	Minimum	Maximum	Standard Deviation	p value
PG	40.67	20	46	7.06	<0.001*
CG	45.82	43	46	0.67	

OCB: Observation of Communicative Behavior; PG: Preterm Group; CG: Comparison Group. Mann-Whitney Test. Significance level of p<0.05. *: Significant values

**Table 2:** Results of the ABFW instrument of the PG compared to the CG

ABFW	Group	Mean	Minimum	Maximum	Standard Deviation	p value
UVD	EG	72.46	27.16	96.01	17.51	0.00*
	CG	86.84	69.3	95.74	5.86	
RP	EG	17.4	4.33	35.17	7.74	0.00*
	CG	11.6	4.24	22.56	4.45	
ND	EG	9.85	0	48.44	12.42	0.00*
	CG	1.5	0	8.12	1.89	

PG: Preterm Group. CG: Comparison Group. UVD: correct appointment; RP: replacement processes; ND: no designation. Student "t" test. Significance level of p<0.05. *: Significant values

**Table 3:** Results of the PPVT instrument of the PG compared to the CG

PPVT	Group	Mean	Minimum	Maximum	Standard Deviation	p value
	EG	106.82	0	127	20.98	0.00*
	CG	119.05	99	139	11.51	

PPVT: Peabody Picture Vocabulary Test.PG: Preterm Group. CG: Comparison Group. Mann-Whitney Test. Significance level of p<0.05. *: Significant values

**Table 4:** Results of the MCDI instrument of the PG compared to the CG, parental report and teachers report

MacArthur	Group	Mean	Minimum	Maximum	Standard Deviation	p value
**Parents**						
Receptive Vocabulary	PG	95.45	90.88	100	0.71	0.99
	CG	99.99	99.77	100	0.03	
Expressive Vocabulary	PG	93.17	34.71	100	15.83	<0.001*
	CG	99.88	97.06	100	0.46	
**Teachers**						
Receptive Vocabulary	PG	90.49	85.69	100	2.36	0.68
	CG	99.99	99.77	100	0.05	
Expressive Vocabulary	PG	89.97	34.61	100	17.97	<0.001*
	CG	99.28	85.36	100	2.37	

MCDI: MacArthur Communicative Development Inventory. PG: Preterm Group. CP: Comparison Group. Mann-Whitney Test. Significance level of p<0.05. *: Significant values

The descriptive analysis of the categories evaluated by the OCB found that 45% of the children from the PG had difficulties to participate in and maintain dialogic and narrative activities; 42.5% had attention difficulties; 30% start and respect the turn change; 25% production of sentences; 22.5% interaction with the evaluator and communicative intention; and oral productions and production of words; 17.5% eye contact, protest, request and offer; 15% report and imitate. In the CG, 5% had difficulties in the narrative category and maintenance of dialogic activities.

**Figure 2:**
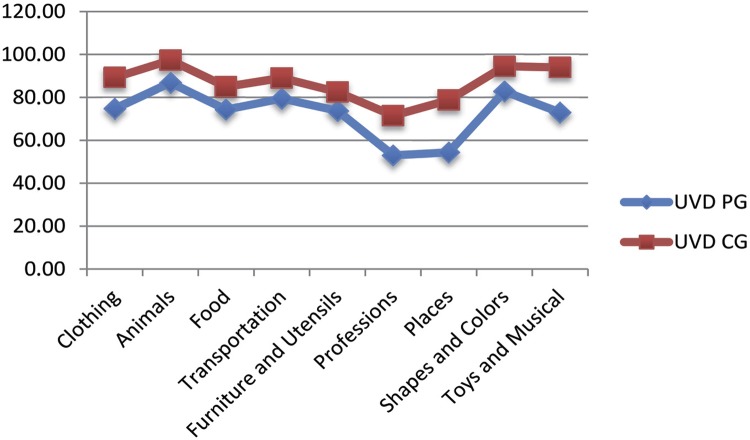
Performance of the Usual Verbal Designation (UVD) of the ABFW instrument in the PG compared to the CG

PG: Preterm Group; CG: Comparison Group; UVD: correct appointment; RP: replacement processes; ND: no designation. Student’s t-test. Significance level of p<0.05. *: Significant values

Figure 2 shows the average performance of the Usual Verbal Designation (UVD) of the PG and CG in the ABFW- Vocabulary Test in each category evaluated: 1- Clothing; 2- Animals; 3- Food; 4- Means of transportation; 5- Furniture and utensils; 6- Professions; 7- Places; 8- Shapes and colors; 9- Toys and tools.

Since it refers to the receptive vocabulary, statistically significant differences were not observed, but the expressive vocabulary presented statistically significant differences for both parents and teachers.

**Figure 3:**
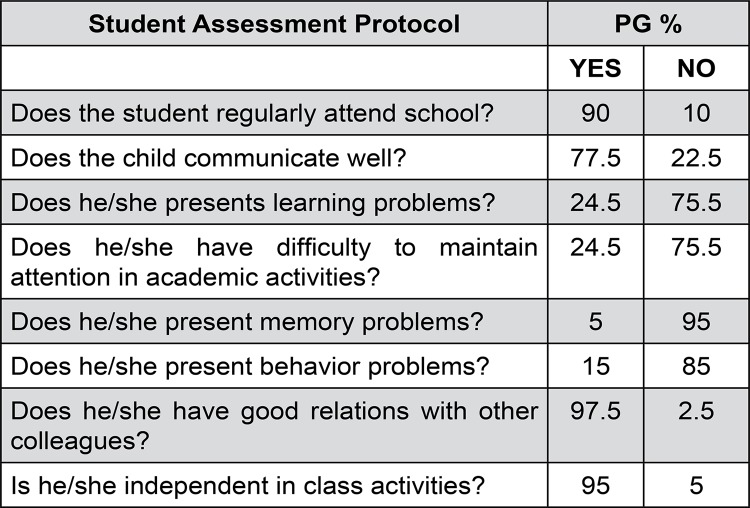
Results of the Student Assessment Protocol of the PG and CG

Figure 3 shows the data obtained in the Student Assessment Protocol for the PG, we note that no information was considered relevant when compared to the CG.

## Discussion

The objective of this study was to verify the performance of premature infants without neurological impairment that were not identified with developmental problems, despite the risk condition of prematurity. Literature suggests that preterm infants without major neurological impairment may have milder functional problems which often are not diagnosed until school age^6^. There are also subtle problems that can relate to communication skills, causing poor performance in language levels.

Regarding the Observation of Communicative Behavior (Table 1), the categories with the highest losses were: narrative, maintaining dialogic activities and attention difficulties. Starting dialogue, maintaining it and performing narrative communicative activities are relevant for the child to integrate in social environments. These are complex skills constructed throughout child development. These activities depend on intrinsic conditions such as brain maturation, perceptual processing, involving attention, discrimination, memory, analysis and synthesis and motivation, but also on extrinsic influences related to the stimuli received from the sociocultural environment.

The narrative of a story in oral or written form refers to the presentation of a series of events in temporal, fictional or factual order^8^. Narrative requires a more complex style of language, which is necessary for academic success, since it involves verbal skills such as the vocabulary and syntactic organization to produce tense and phrasal organization according to the language, factual memory and reasoning, among other perceptual skills^8^ ^,^ ^21^. Thus, the narrative is considered a good predictor of academic success^3^. Studies showed that preterm infants have higher chances of difficulties in tasks involving more complex language skills^8^ ^,^ ^21^.

The difficulty to maintain attention was a category in which many of the participants of the PG had difficulties. Attention involves a series of processes (or subcomponents) that allow us to focus and select a conscious event, being dependent on systems and anatomical subsystems working in parallel as networks, which allow the processing of concurrent and interactive information, favoring performance in cognitive and linguistic tasks interfering with daily activities and the development of learning processes, as well as maintaining dialogic activities and narrative.

O’Shea, Downey and Kuban^24^ (2013), reported an association between prematurity and especially extreme prematurity with the type of inattention disorder of attention deficit hyperactivity disorder. The impairment of attention among the premature affects a range of attention areas including selective attention and sustained attention, being identified mainly at school age by social demands and due to the interference of this ability in academic success. Other studies link attention changes as interfering in the learning process of premature infants^5^ ^,^ ^13^ ^,^ ^24^.

Expressive vocabulary was assessed through the ABFW instrument (Table 2 and Figure 2). For UVD, the PG and CG obtained higher scores, respectively, in the categories: animals; shapes and colors; means of transportation; clothing; foods; furniture and utensils; toys and instruments; places and professions.

According to a meta-analysis study on the performance of preterm infants in language, premature and very low birth weight children had the worst performance in vocabulary, considering the semantic subdomains^8^.

The acquisition of vocabulary is related to the experience of the child in the social environment. We infer that the highest mean obtained by the categories animals and shapes and colors is related to school experiences. Shapes and colors are contents of preschool learning and animals are often reported in children stories. Regarding the furniture and utensils category, we expected good performance from the children because of daily activities, however this does not always occur, probably because these two categories are grouped by the instrument, namely, children showed greater knowledge of items such as a cup, plate, spoon, pot, but had greater difficulties in the furniture category, such as chest, ironing table and clothes, etc. The least popular categories were professions and places.

Regarding the replacement processes (RP), when participants in the CG were not certain of their answers, they often perform replacement processes of words, usually in the same semantic classification or designated function, for example, “pineapple” is replaced by “fruit”. Participants of the PG often did not designate and did not present verbal labels that demonstrate content knowledge. We infer that in these cases, the replacement process is a development in communication skills, since the answers given, although qualified as replacement processes, are related with the expected. In conversational situations this could facilitate their communication interaction with their interlocutor.

Guarini, et al.^11^ (2010), reported that when compared to children born at term, premature children presented smaller development on vocabulary, grammatical and phonological awareness and language skills, even those without brain injury. For Marchman, et al.^23^ (2016), individual differences in the efficiency of lexical processing may be able to serve as a marker for information processing skills that are critical for language learning.

The environment can favor receptive development and expand the vocabulary and its use, i.e. the family, school and other social environments require the use of more elaborate linguistic content, and the child will create opportunities not only to acquire verbal labels, but also to expand his/her linguistic structures, making him/her an effective communicator (according to his/her capacities).

Several authors reported that premature babies can have problems in lexical acquisition and describe factors that can influence the acquisition of vocabulary, such as the environment, sex, socioeconomic status, maternal education and pregnancy complications^7^ ^,^ ^16^ ^,^ ^18^ ^-^ ^22^.

Regarding the socioeconomic status and maternal education, some studies indicate that these variables are very important for the development of language^14^ ^,^ ^20^ ^,^ ^28^. However, child development occurs with the exposure of the child to different social environments and, more important than maternal education is the degree of interaction of the child with the mother^20^. We note that the verification of the socio-economic level by the instrument used considers maternal education as one of the criteria^27^. The groups of this study were matched by sex, chronological age (in months), education, type of school (public or private) and socioeconomic status. This could approximate the groups.

Several studies used the PPVT as an instrument to measure the receptive language of preterm infants^1^ ^,^ ^5^ ^,^ ^20^ ^,^ ^31^. Luu, et al.^21^ (2009) performed studies with extremely premature newborns in a longitudinal follow-up. Caravale, et al.^5^ (2012) assessed children with low neurologic risk. Adams-Chapman, et al.^1^ (2015) assessed children with extremely low birth weight and Marchman, et al.^23^ (2016) assessed children born of gestational age under 32 weeks and low weight. Van Noort-van der Spek, Franken and Weisglas-Kuperus^31^ (2012) performed meta-analyses of studies that used the PPVT and found 13 studies indicating that premature babies showed receptive vocabulary not as developed as children born at term.

Receptive and expressive vocabulary (Table 4) were analyzed according to the reports of parents and teachers. Individuals of the PG presented more modest scores in receptive vocabulary, with no statistically significant difference. Despite the difficulties in receptive vocabulary, as reported by the application of the PPVT, when parents and teachers were questioned about both receptive and expressive vocabulary, they only reported difficulties in expressive vocabulary. Some studies have used the MCDI instrument to verify the receptive and expressive vocabulary^7^ ^,^ ^18^ ^,^ ^28^.

The participation of the family in understanding the communication skills of their children is extremely important since they can analyze the receptive and expressive possibilities by observing the activities of daily living. The same argument can be made for the participation of teachers due to the daily coexistence in the classroom. Thus, it is critical for parents and teachers to understand the nature of the deficits that will interfere on the academic performance so these children are identified early, which favors the management of the difficulties and reduces the impact on school learning^15^ ^,^ ^24^.

The children participating in this study are in the pre-literacy phase, since in Brazil, the literacy period begins at age six. In the study by Kessel-Feddema, et al.^18^ (2007), 37% of students born premature had learning difficulties. Other difficulties were identified in the observation of teachers such as communication difficulties and problems with attention that interfere on academic learning, especially in the literacy phase.

Literature shows, over time, that children born prematurely are at risk for learning difficulties^12^ ^,^ ^13^ ^,^ ^15^ ^,^ ^17^ ^,^ ^23^ ^-^ ^25^. Adverse birth conditions are strongly associated with academic learning problems, and prematurity is studied as one of the most common risk conditions for the development of academic skills.

Thus, identifying the areas of readiness for educational success is extremely important. Pritchard, et al.^26^ (2014), showed that the academic performance of these children remains poorly understood and even less is known about the best strategy to identify those at higher risk. Identifying the difficulties during preschool may favor the therapeutic procedures that support these children on their transition from preschool to literacy.

Children born prematurely with low risk of neurological sequelae in preschool age may have greater difficulties than their peers born to term, this should be known by teachers and families so they can interfere in this and facilitate the integration and learning of the children at school^30^. Thus, all preterm infants should participate in long-term monitoring programs to track their development. Literature also points to this need^7^, allowing preventive procedures or timely rehabilitation procedures to avoid learning difficulties at school age.

Thus, this study provides reflections that can promote understanding of the performance of preterm children, such as their receptive and expressive vocabulary, since they contribute to the communicative and educational development, integrating these individuals with their age group and encouraging the development of important skills for literacy.

The generalization of research data is hindered by the methodological differences of studies, such as cohort differences, eligibility criteria for samples, native language, cultural differences, instruments used and heterogeneity of the population of premature infants, regarding both biomedical and environmental factors. However, the risks of change in the acquisition and development of language should be considered.

We note that our sample was restricted, and in the training of the experimental group we did not consider the classification according to the gestational age (extreme moderate or late) or birth weight, but sought infants born preterm without neurological damage and complaints of developmental disorders.

We reiterate that future studies should follow the overall development of premature infants longitudinally, to contribute to the acquisition and development of skills that will promote performance in communicative activities and learning, as well as to invest in the best indicators to monitor the specific language skills throughout the school years of premature children.

Some findings of this study seem to be congruent, such as the influence of prematurity on child development, and especially the need for family counseling for the observation of child performance and school attention to prevent the deleterious effects of prematurity during his/her development and school life.

One contribution of this study was to show that premature children may have problems in the acquisition of receptive and expressive vocabulary, even in the absence of neurological dysfunctions and family complaint. We note that in this sample the mothers did not present complaints of language alterations, however, in the statistical analysis there was a statistically significant difference for both receptive and expressive language. This study intends to contribute so the professionals who work with these children can be attentive to the performance in language tests of children born prematurely, even for those children who initially do not present complaints of delay in language development.

## Conclusion

Children born prematurely with low risk of neurological sequelae in preschool age may have greater difficulties in linguistic performance when compared to their peers born to term.
